# Would Stop the Medical Steam Roller

**Published:** 1911-02

**Authors:** 


WOULD STOP THE MEDICAL STEAM ROLLER.—Our esteemed friend and colleague, Dr. M. M. Smith of the *Texas Medical News* (Dallas, January number), advises that some changes be made in the conduct of the House of Delegates of the Texas State Medical Association.

Try it.

Yours truly had such Utopian ideas once,—and at Dallas he brought up an amendment of the Constitution forbidding negroes becoming members; a resolution separating the annual dues from the journal subscription, as required by law; a resolution to print the valuable papers in separate volume, intending to give the "scientific body," *the ninety-five per cent of disfranchised members, the benefit of the constitutional right to vote on such measures.* These resolutions were introduced the first morning. They were at once sidetracked; referred to a committee of three, appointed by President Russ, and this committee reported adversely,—*on the afternoon of the last day,*—too late for any action by the majority, the disfranchised,—who, "like dumb-driven cattle," tamely submitted. Moral: Don't monkey with the buzz-saw. Try it, Matt, and see how good it feels to get run over.

